# CYClones: a highly powered, fully genotyped, eight-parent yeast mapping population

**DOI:** 10.1093/g3journal/jkag179

**Published:** 2026-07-03

**Authors:** Gareth A Cromie, Russell S Lo, Trey S Morgan, Anne E Clark, Julee Ashmead, Martin S Timour, Amy Sirr, Joshua M Akey, Aimée M Dudley

**Affiliations:** Pacific Northwest Research Institute, Seattle, WA 98122, United States; Pacific Northwest Research Institute, Seattle, WA 98122, United States; Pacific Northwest Research Institute, Seattle, WA 98122, United States; Department of Genome Sciences, University of Washington, Seattle, WA 98195, United States; Pacific Northwest Research Institute, Seattle, WA 98122, United States; Pacific Northwest Research Institute, Seattle, WA 98122, United States; Pacific Northwest Research Institute, Seattle, WA 98122, United States; Lewis Sigler Institute, Princeton University, Princeton, NJ 08540, United States; Department of Ecology and Evolutionary Biology, Princeton University, Princeton, NJ 08544, United States; Pacific Northwest Research Institute, Seattle, WA 98122, United States

**Keywords:** Multiparent advanced generation intercross, Mapping population, Funnel cross, Budding yeast

## Abstract

The budding yeast *Saccharomyces cerevisiae* is a remarkably adaptable organism that thrives in diverse environments. Global sequencing of natural isolates has revealed extensive genetic diversity within the species. Here, we describe the construction and characterization of CYClones (Collaborative Yeast Cross clones), a library of 11,392 segregants generated from a multiparent funnel cross of eight genetically diverse parental strains. To enable the genetic dissection of complex traits, we imputed whole-genome sequences for all segregants and show that CYClones captures a substantial fraction of the global genetic diversity of *S. cerevisiae*. Haplotype representation is well maintained, with each parental haplotype present at >5% frequency across >95% of the genome. Simulations demonstrate that CYClones has ≥95% power to detect variants with heritability as low as 0.36%, with mapping resolution often finer than the length of a single gene. In summary, CYClones is a powerful community resource for dissecting the genetic architecture of complex and quantitative traits, uncovering context-dependent mutational effects, and identifying causal variants underlying phenotypic diversity.

## Teaser

This work is aimed at researchers studying how genetic differences influence traits in yeast. Cromie et al. present CYClones, a large, fully genotyped population of over 11,000 yeast strains derived from eight diverse parents. This resource captures a substantial portion of the genetic diversity found in natural isolates while reducing confounding effects of population structure. It enables precise mapping of genetic variants, including those with small effects, and supports localization of causal variation at fine, sometimes single-gene, resolution. CYClones provides a powerful and flexible tool for studying complex traits and gene interactions in yeast.

## Introduction

The budding yeast *Saccharomyces cerevisiae* was the first eukaryote to have its genome fully sequenced (Goffeau et al. 1996; Williams 1996), and as sequencing costs have plummeted, the yeast community has characterized large collections of natural variant strains. Because *S. cerevisiae* has been associated with human food and beverage production for at least 7,000 years (McGovern et al. 2004; Liu et al. 2019), it has been exposed to an unusually broad range of environments. Sequenced isolates now span human-associated products (wine, beer, sake, coffee, cacao); natural sources such as tree bark, soil, and insects; and human clinical subjects (Ezov et al. 2006; Goddard et al. 2010; Wang et al. 2012; Angebault et al. 2013; Cromie et al. 2013; Skelly et al. 2013; Strope et al. 2015; Gayevskiy et al. 2016; Ludlow et al. 2016; Zhu et al. 2016; Peter et al. 2018; Fay et al. 2019). These collections have greatly expanded our understanding of the evolutionary history, origins, and population structure of the species (Fay and Benavides 2005; Ronald and Akey 2007; Liti et al. 2009; Schacherer et al. 2009; Fay 2013; Duan et al. 2018).

The genetic variation present in natural populations represents a powerful tool for interrogating genotype–phenotype relationships. Genome-wide association studies (GWAS) capture this variation directly by drawing samples from the natural population (Abdellaoui et al. 2023) and have been increasingly applied to *S. cerevisiae* as comprehensive natural-isolate sequencing efforts have matured (Liti et al. 2009; Schacherer et al. 2009). Despite the species' complex population structure (Connelly and Akey 2012), the 1,011-genome panel of Peter *et al.* (Peter et al. 2018) demonstrated that yeast GWAS is feasible and enabled GWAS across 36 traits. Subsequent panels have extended both scale and resolution: a unified reanalysis of 3,034 isolates compiled across 29 prior studies provides an integrated view of natural genetic and ploidy variation (Loegler et al. 2024), and near telomere-to-telomere assemblies of 1,086 isolates have shown that incorporating structural variants and short indels alongside SNVs improves heritability estimates over SNV-only models (Loegler et al. 2025). However, several intrinsic limitations of natural-population GWAS persist regardless of panel size: population structure remains a confounding factor even after correction, linkage disequilibrium from clonal history limits the resolution at which causal variants can be localized, and the impact of rare variants (with minor allele frequency [MAF] below ∼5%), which may contribute disproportionately to phenotypic variance in yeast (Bloom et al. 2019; Fournier et al. 2019), remains difficult to assess at typical sample sizes.

Mapping populations generated through crossing and analyzed by linkage analysis represent a complementary approach to GWAS. These populations can be generated from two (biparental) or more (multiparent) founders. Yeast biparental crosses have produced influential large-scale QTL studies, most notably the BYxRM segregant panel (Brem et al. 2002; Bloom et al. 2013) and the related X-QTL pooled-segregant approach (Ehrenreich et al. 2010), which together established that yeast complex traits typically have polygenic architectures with predominantly additive per-locus effects (Bloom et al. 2015). To extend the allelic diversity beyond two founders, several yeast multiparent populations have been constructed: the four-parent SGRP-4X advanced intercross provided proof-of-principle at modest scale (Cubillos et al. 2013), a 16-founder round-robin cross of approximately 14,000 segregants demonstrated that rare variants in the founder pool can contribute disproportionately to phenotypic variance (Bloom et al. 2019), and two synthetic 18-way outcrossed populations carried through 12 generations of random mating maintained haplotype contributions from at least 10 of the 18 founders across more than 98% of the genome (Linder et al. 2020). Relative to biparental designs, multiparent crosses bring more alleles into a single experiment and capture rare (in the natural population) alleles at frequencies comparable to common alleles, improving statistical power for rare-variant detection although, as analyses of the Drosophila Synthetic Population Resource have shown, per-variant detection power still depends on MAF in any cross (King, Macdonald, et al. 2012). Multiparent crosses also greatly reduce the population–structure confounding that complicates GWAS, although design choices such as small intermediate pool sizes can introduce other forms of structure, including bottlenecks at intermediate cross stages (Huang et al. 2015).

**Fig. 1.**
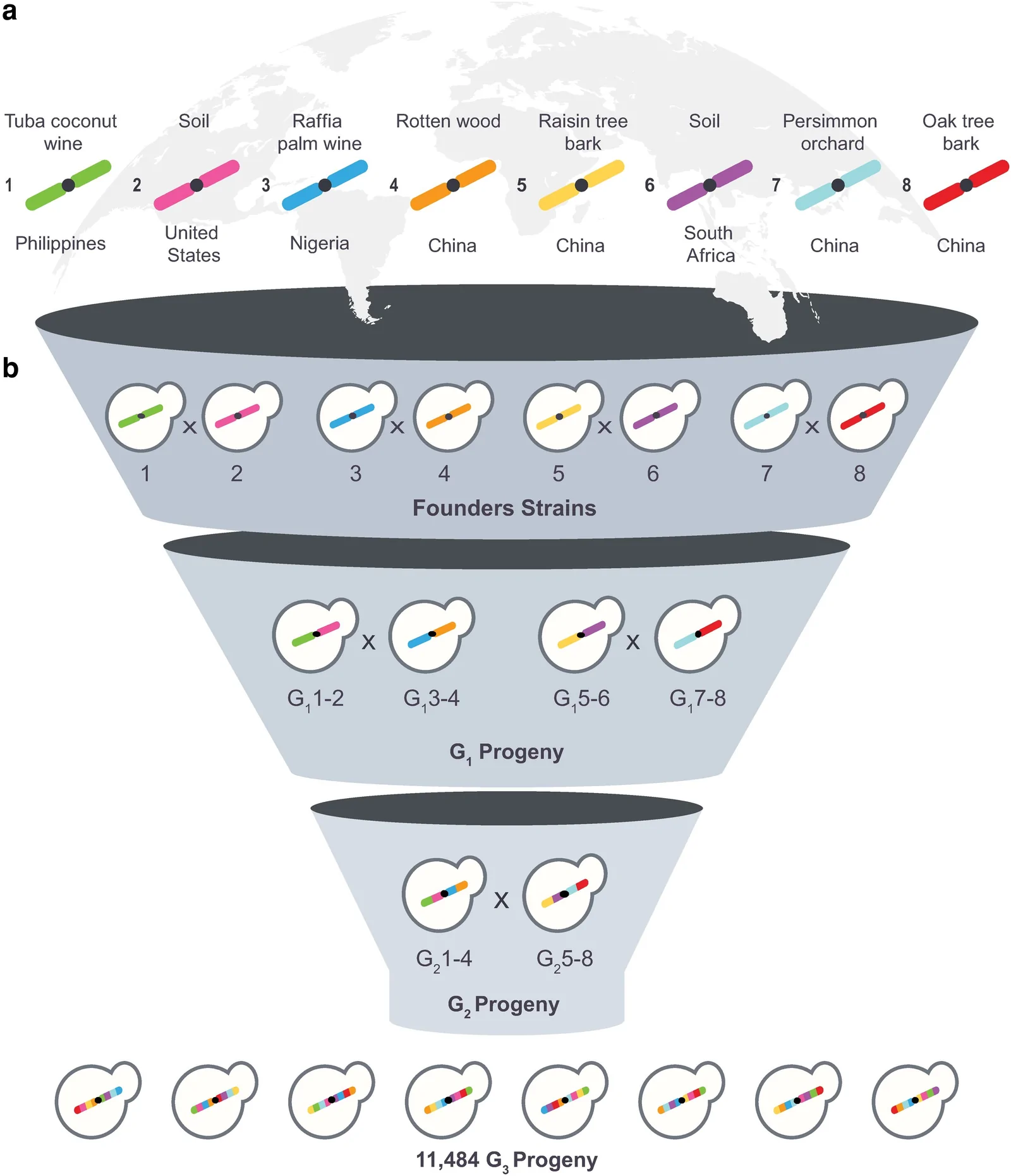
Generation of the mapping population. a) Eight haploid strains from diverse geographical and environmental sources, harboring a substantial proportion of the rare and common alleles present in the global *S. cerevisiae* population, were chosen to generate a multiparent mapping population. b) The mapping population was constructed using a funnel cross design. Each founder strain was mated pairwise to produce pools of haploid progeny. Two rounds of reciprocal crossing using pooled strains were then carried out to produce the final mapping population of ∼11,400 haploid strains, each a recombinant mosaic of the eight founder haplotypes.

We set out to develop a library of yeast strains that captures a substantial fraction of genomic variation in yeast and that avoids the confounding effects of population structure. Multiparent “funnel cross” breeding schemes generate populations that harbor all combinations of parental alleles and have been proposed as an efficient means of accomplishing these goals (Valdar et al. 2006; Rockman and Kruglyak 2008). Therefore, we constructed CYClones (Collaborative Y east C ross clones), a set of 11,392 fully genotyped recombinant strains generated from eight parental yeast strains using a funnel cross design. C ollaborative Y east C ross clones complements complex crosses in other model organisms (generally referred to as M ultiparent A dvanced G eneration I nter-Cross lines), such as MAGIC (Cavanagh et al. 2008; Kover et al. 2009) in *Arabidopsis*, the Drosophila Synthetic Population Resource (Huang et al. 2012; King, Merkes, et al. 2012) in *Drosophila*, and the mouse collaborative cross (Churchill et al. 2004). Indeed, our eight-parent funnel cross in the budding yeast, *Saccharomyces cerevisiae* (Fig. 1), was inspired by the mouse collaborative cross (Churchill et al. 2004), which aimed to generate 1,000 recombinant inbred lines derived from eight parental mouse strains. However, since that project was hampered by exceptionally high (95%) extinction of the recombinant inbred lines during the final inbreeding process (Churchill et al. 2004), a large, highly powered multiparent mapping population generated via such a design has yet to be examined.

## Materials and methods

### Media and genetic manipulation of yeast

Unless noted, standard media and methods were used for the growth and manipulation of yeast (Rose et al. 1990). The *S. cerevisiae* founder strains used in this study are listed in Table 1; the strains used in their construction are in Supplementary Table 1. Unless specified, yeast strains were grown in YPD medium (1% yeast extract, 2% bacto peptone, 2% glucose) with 2% agar for solid plates. Nourseothricin (NAT) was used at a final concentration of 100 µg/ml. Hygromycin (HYG) was used at a final concentration of 500 µg/ml.

### Founder whole-genome sequencing, assessment of global yeast variation captured, and phylogenetic tree construction

The genome sequences of each of the eight haploid founder strains used to construct the mapping population were determined as follows. Genomic DNA was isolated using a YeaStar Genomic DNA Extraction Kit (Zymo Research). DNA sequencing libraries were prepared using the Paired-End Sequencing Kit (Illumina) following the manufacturer's instructions. Sequencing reads were generated on an Illumina NextSeq 500, with 38 + 37 bp paired-end reads for all strains, except YO2685 for which 43 + 32 bp paired-end reads were used. Adaptor sequences were trimmed using Cutadapt (v1.7.1) (Martin 2011). Sequences were then aligned to the S288c reference genome (R64-1-1) using BWA (v7.9) (Li and Durbin 2009), using the “aln” option with quality trimming (threshold of Phred = 20) and allowing a maximum of six mismatches. PCR duplicates were removed using the SAMtools (v0.1.19) (Li et al. 2009) “rmdup” command. BCFtools (v1.10.2) (Li et al. 2009) was then used to generate a variant call file (vcf) for each parental strain, using the “mpileup” command with the parameter “-d 10000” and the “call” command with the “-c” flag (consensus caller).

The VCF files were then assessed at all biallelic single nucleotide variant (SNV) sites with <5% missing data identified in a genomic analysis of the global yeast population (Peter et al. 2018). The proportion of global sites for which we identified both alleles among our founder strains was then calculated for a range of MAF cutoffs in the global dataset. For phylogenetic tree construction, the set of sites was then further reduced to only those with quality scores >1000 in the published global gVCF file (Peter et al. 2018). The haploid founder strains were added to this dataset of global yeast strains, encoded as homozygous reference, homozygous alternative, or missing data at each site. A neighbor-joining tree was then generated using the dissimilarity distance, as described in Peter *et al.* (Peter et al. 2018).

### Identification of SNVs among the founder strains

The sequencing reads for each founder strain were also used to generate a *de novo* genome assembly for that strain, and this assembly was compared to the reference genome to identify SNVs. First, reads were error-corrected with SGA (v0.9.9) (Simpson and Durbin 2012) using the “preprocess” command with the parameters “-m 25 -p 1 -q 10 –permute-ambiguous” and were then corrected using the “correct” command with the parameter “-k 25.” The corrected reads were assembled into contigs using IDBA (v1.0.12) (Peng et al. 2012) with the parameters “–mink 19 –maxk 35 –step 2 –seed_kmer 23.” The final contigs were aligned to the reference genome using the Mummer (v 3.23) “nucmer” command with the parameters “-c 100 -l 10 -b 200,” and the alignments were then filtered using the “delta-filter” command and the parameters “-r –q.” Finally, bases were called in the aligned regions using the “show-SNVs” command.

A set of 276,774 high-quality SNVs segregating in the mapping population was then identified by combining the VCF files from each founder strain with the basecalls from the *de novo* assembly pipeline (File #1 in https://doi.org/10.5281/zenodo.17362653). Only biallelic SNVs with basecalls in all founder strains, consistent in both the VCF and *de novo* files for each strain, were accepted. In each VCF file, a basecall quality cutoff of greater than or equal to 50 was also applied, and, for non-reference calls, the homozygous model for that basecall had to be more probable than the homozygous reference or heterozygous models.

### T2T founder assemblies and structural variation resource

As a downstream resource for causative-variant follow-up, near telomere-to-telomere (T2T) assemblies of the eight founder strains were generated by Oxford Nanopore long-read sequencing on R10.4.1 flow cells (Plasmidsaurus, Eugene OR; commercial service, 2023). Reads were basecalled with the Dorado superaccurate r1041_e82_400bps_sup_v4.2.0 model, assembled with Flye 2.9.2, and polished with Medaka 1.8.0. Each polished assembly was aligned to S288c (R64-1-1); SNVs and small indels (<50 bp) were called with minimap2 (v2.28, -cx asm5 –eqx) and paftools.js and structural variants (≥50 bp) with MUMmer4 (nucmer –maxmatch -c 100 -l 50) and MUM&Co, following the conventions of recent yeast pangenome resources (Loegler et al. 2025). A per-founder SyRI overlay on the same minimap2 alignments was generated alongside the MUM&Co calls.

The variant calls from this pipeline were not used to generate the mapping marker set described in this study; the founder marker set was generated from short-read sequencing of the founders (above), matching the short-read sequencing used to genotype the progeny strains. Concordance of the T2T pipeline against the short-read marker set was assessed using the per-founder minimap2 + paftools.js SNV calls (the < STRAIN>.snv_indel.lt50.vcf.gz files; minimap2 -ax asm5 of each scaffolded founder assembly against S288c R64-1-1, paftools.js call with -L 10000 -l 1000 -q 5, filtered to variants <50 bp). Marker–position recall, the fraction of marker–set variant calls (pooled across the eight founders) reproduced with the matching alt allele by the T2T pipeline, is 96.5% genome-wide (443,206/459,069 marker–set variant calls) and 98.0% when variants in the outer 30 kb of each chromosome (subtelomeric regions) are excluded. When both pipelines call a variant at a shared position, allele agreement is 99.999% (four disagreements in 443,210 shared calls). Per-founder and multi-sample VCFs, the assemblies, the bash workflow scripts (setup.sh, run_all.sh, run_one_strain.sh), and the concordance script that produced these recall numbers (concordance_marker_vs_t2t.py) are bundled as File #9 in https://doi.org/10.5281/zenodo.17362653.

### Constructing the mapping population

To prevent mating type switching, and thus maintaining stable haploid strains at each round of the cross, *hoΔ0* haploid founder strains were generated from the original diploid strains (Supplementary Table 1) as follows. First, in each diploid strain, one copy of the gene encoding the HO endonuclease was deleted by integration of an HO-hisG-NatMX6-pGPD-yeGFP-tURA3-hisG-HO fragment excised from the pTM10-HO_GFP (AB465) plasmid (GenBank: OM069737). These diploids were then sporulated, tetrads were dissected, GFP^+^/NAT^R^ haploid progenies were identified, and their mating types were determined by PCR (Huxley et al. 1990). These isolates were then propagated on YPD, during which a loop-out of the HO–cassette can occur at a low rate via homologous recombination of the hisG direct repeat. These homologous recombination events leave behind a single copy of the (1130 bp) hisG sequence at the locus but remove GFP and the NAT drug marker, permitting the future use of these markers. Cells having undergone the desired recombination events were isolated as GFP^−^ cells by FACS, confirmed by PCR using primers AO34 (CTTTGTATCCTTAATAAAATAAAATTCACA) and AO579 (TAAATAGTTACCACAAGGCCATATC) and then tested for NAT sensitivity.

To facilitate mating and determination of progeny mating type, one of two drug markers (hphMX or natMX) (Goldstein and McCusker 1999) was integrated in the founder strains between *PHO87* and ARS314 (position 197,356 on Chromosome III), which is close to the mating type locus. As a result, in the haploid founder and progeny strains at all rounds of the cross, MATa haploids harbored the HYG B phosphotransferase (*HPH*) gene conferring HYG resistance, while MATα haploids harbored the NAT N-acetyl transferase (*NAT*) gene conferring NAT resistance. These drug resistance phenotypes were used to distinguish diploids and haploids of each mating type. For the final set of eight founder strains, MATa isolates were chosen for four strains (Founders 1, 3, 5, and 7), and MATα isolates were chosen for the remaining four (Founders 2, 4, 6, and 8).

The mapping population was then derived from the eight founder haploids through three consecutive rounds of pooled mating and sporulation in a “funnel” design (Fig. 1b and Supplementary Fig. 1) as follows. For the initial cross, matings were performed in liquid by mixing 150 µl of overnight YPD cultures, each seeded with a single haploid parental colony. The mixtures were incubated at 30 °C for 5 h, without shaking, and then plated on YPD + HYG + NAT agar. A single diploid colony was chosen for further crossing. The second and third rounds of mating were performed in liquid as above, using two pools of haploid progeny from the previous cross. Specifically, 150 µl of YPD supplemented with the appropriate drug selection was seeded with individual haploid segregants (*n* = 576 strains/mating type) and grown overnight. 10 µl aliquots were then pooled and washed to remove the selective drug. The washed pools of the two haploid populations were mixed and plated directly onto YPD agar. Plates were grown overnight at 30 °C, forming lawns which were scraped, washed, and then diluted into YPD + HYG + NAT liquid medium and grown, to select for diploids. For all rounds of the cross, heterozygous diploids were grown in YPD + HYG + NAT liquid medium to exponential phase, washed, and then sporulated in Tong and Boone enriched sporulation media (Tong and Boone 2006). Once sporulated, tetrads were disrupted and individual haploid recombinant progeny were isolated using a FACS-based method previously described (Sirr et al. 2022). Briefly, 1 ml of the culture was stained with 1 µg/mL DiBAC_4_(5) [bis-(1,3-dibutylbarbituric acid) pentamethine oxonol] (AAT Bioquest 21410), a fluorescent vital dye which selectively stains tetrads and dead cells. To digest the ascus surrounding each tetrad, stained cells were treated with zymolyase 100T, and tetrads were disrupted using sterile 0.5 mm diameter glass beads (Biospec Products CAT#11079105) resulting in a population of single spores with very few dyads, triads, or intact tetrads. A population of single spores was identified using a Sony LE-SH800 cell sorter configured with a 100 µm microfluidic sorting chip, the 488 nm laser for FSC/BSC detection, and the 561 nm laser and 617/30 nm bandpass filter set for detecting fluorescence. Individual spores were sorted using the Ultra-Pure setting into single wells of a 96-well plate containing liquid YPD. After growth in YPD, each sorted plate was then replica pinned to both YPD + HYG (to identify MAT**a** cells) and YPD + NAT (to identify MATα cells). Strains that were resistant to both NAT and HYG were discarded.

A final set of 5,742 MAT**a** haploid cells and 5,742 MATα haploid cells were collected at the end of the funnel process, for a total mapping population of 11,484 haploid strains. Strains were stored in a 15% glycerol solution at −80 °C in 96-well plates, one strain per well.

### Determining ploidy and haplotype of progeny strains

Haplotypes of the 11,484 haploid progeny in the final round of the funnel cross were inferred based on low-coverage (median 3.8-fold) WGS as follows. Note that a total of 92 strains failed sequencing quality control, resulting in the final population of 11,392 fully haplotyped strains. Genomic DNA was extracted in 96-well plate format by the method of Drumonde-Neves *et al.* (Drumonde-Neves et al. 2013) and quantitated via a SYBR Green/spectrophotometer assay (Leggate et al. 2006) to obtain a plate-averaged DNA concentration which was then used to dilute the plate concentration to a range optimized for the PlexWell library prep (target = 10 ng template DNA per library prep reaction). Libraries were prepared using the PlexWell 384 Library Preparation Kit (SeqWell Inc., Beverly, MA) according to the instructions in User Guide v20190813. Using this method, each strain was first individually tagged with P7 primers containing a well-specific i7 index using a limited amount of transposase, which serves to normalize the samples. Then, wells of a plate were pooled and tagged using a transposase with a P5 primer containing a plate-specific i5 barcode. Finally, the pool was magnetic bead-purified and PCR-amplified to enrich P7-/P5-tagged fragments and to create the multiplexed library for Illumina sequencing. Paired-end (75 + 75 bp, except for 348 strains with 38 + 37 bp) library sequencing was then performed on an Illumina NextSeq 500.

After demultiplexing, sequencing reads underwent adaptor trimming and were aligned to the S288c reference genome as described above for sequencing of the founder strains. A pileup file was then produced for each strain, using the SAMtools (v0.1.19) “mpileup” command with the -C 50 parameter and the BCFtools (v 0.1.17) “view” command with the -c flag (consensus caller). The full set of 11,484 strains was then filtered to remove any with low sequencing coverage (≤12k reads, approximately 0.1-fold coverage), leaving 11,392 sequenced strains. Mean sequencing depth per chromosome was used to estimate chromosome copy number and to identify aneuploid strains (Supplementary Table 2).

For each strain in this set, at each position identified in the founder biallelic SNV table, the number of reads matching each of the two alleles was counted from the pileup file for that strain. These counts (Files #2 and #3 in https://doi.org/10.5281/zenodo.17362653) were then used to infer the haplotype state of the strain at each of the SNV positions by fitting a hidden Markov model (HMM) to the allele counts using the Viterbi algorithm. The HMM had eight states, corresponding to the eight founder haplotypes. At each SNV site, the emission probabilities for each haplotype were set to 0.99 for the expected allele from that founder strain, with the incorrect allele probability set at 0.01 (error probability). The transition probabilities between haplotype states at each adjacent pair of SNV sites were calculated by combining the expected recombinant fraction per meiosis (R) with the structure of the cross. Specifically, for each pair of adjacent SNVs, R was calculated by first converting the physical distance between the SNVs into genetic distance using a conversion of three crossovers per megabase (Mancera et al. 2008). Then, the genetic distance (m) was converted into R using Haldane's mapping function, where R = 0.5x(1-e^−2m^). Finally, the structure of the cross was used to determine the transition probabilities (for chromosomes with copy number 1), as a set of equations based on R. For instance, for two adjacent markers in a mapping population strain to both have Haplotype 1, no crossover can have occurred in the interval between them in any of the three rounds of crossing, so this transition probability is (1-R)^3^. Similarly, for a marker to have Haplotype 1 and its neighbor marker to have Haplotype 2, a crossover must have occurred in the first round of the cross, but no crossover in the remaining two rounds, corresponding to R(1-R)^2^. The full set of equations is given in Supplementary Table 3. The final haplotype inferred for each strain is given in File #4 in https://doi.org/10.5281/zenodo.17362653. A separate set of transition probabilities was used for disomic chromosomes (Supplementary Table 4).

Pooled sequencing and allele counting of intermediate population G_1_5–G_1_6 (Fig. 1b) were carried out as described above for individual mapping strains, but at a median sequencing depth of 392-fold at marker positions.

### Scaffolding marker subset

A subset of markers was chosen to provide a genome-wide “scaffold,” with markers spaced ∼2 kb apart so that causative variants should generally be within 1 kb of a scaffolding marker. This reduces the number of markers at which statistical tests are carried out while maintaining power and resolution. The set of markers was chosen starting with the first marker on each chromosome, taking the first marker ≥2 kb away and repeating until the entire genome was covered. The final scaffold has 5,479 markers (File #5 in https://doi.org/10.5281/zenodo.17362653). We note that this physical distance-based selection does not account for variation in local recombination rates; in regions of low crossing over the scaffold may include more markers than necessary for resolution, and in regions of high recombination a denser scaffold could in principle improve power. However, the genome-wide simulations reported in the Results indicate that the chosen scaffold density is sufficient to retain near-causative marker mapping power in practice (Fig. 5a).

### Identifying mapping strains with shared ancestors in the intermediate funnel cross populations

To assess bottlenecking, we set out to identify mapping strains descended from the same strain in one of the intermediate funnel cross populations. First, for each pair of mapping strains, regions of the genome descended from the same intermediate cross population were identified as follows. Consider shared ancestry in intermediate population G_1_1–G_1_2 (Fig. 1b; equivalent to HA1/HA2 in Supplementary Fig. 1), produced by crossing Founders 1 and 2. Let x and y be the haplotypes of the two mapping strains being compared. Regions with G_1_1–G_1_2 ancestry in both strains are identified as x,y Î {1,2}. For the remaining G_1_ ancestral populations, the test sets used were {3,4}, {5,6}, and {7,8}. Regions with shared G_2_ ancestry in both mapping strains were identified using the test sets {1,2,3,4} and {5,6,7,8} (Fig. 1b).

For each pair of mapping strains and each intermediate population, haplotype discordance was then measured across the shared subset of the genome. Discordance was calculated as the proportion of markers with non-matching haplotypes in these regions. For aneuploid strains, discordance was calculated only using monosomic chromosomes. Discordance values of 0 are expected when strains share a single ancestor in the intermediate population; otherwise, values of 0.5 (G_1_ populations) or 0.75 (G_2_ populations) are expected. To identify groups of strains with a single shared ancestor, hierarchical clustering using the “complete” method was applied to the discordance distances, using the “hclust” command in R.

### Relationship between physical and genetic distances

For two markers on the same chromosome to have the same haplotype value in a mapping population strain, there must have been no net crossing over between them in any of the three rounds of the funnel cross. Therefore, I = (1-R)^3^, where I is the proportion of strains in which the two markers have the same haplotype (identity), and R is the recombinant fraction between those markers per meiosis. In Haldane's mapping formula, R = 0.5x(1-e^−2m^), where m is the genetic distance between two markers. Therefore, I = (1-0.5x(1-e^−2m^))^3^. By comparing the observed value of I to the value predicted by this formula, a single physical-to-genetic distance conversion coefficient was then estimated by maximum likelihood. Specifically, a range of conversion coefficient values was used to predict I from the physical distance between markers. The value that minimized the residual variance after regressing the observed value of I onto the predicted value was then identified.

### Power simulations

To assess the QTL mapping power of the scaffolding marker set, we used independent single-QTL phenotype simulations on the 9,351 euploid mapping strains. A panel of 1,000 causative markers was assembled by sampling 2,000 markers at random from the ∼270k high-quality SNVs, recoding each segregant's haplotype call into a major/minor genotype using the founder allele table, retaining only those markers at which both genotype classes are present in the euploid mapping population (a precondition for the phenotype simulation), and taking the first 1,000 of the remaining set. For each causative marker, one phenotype was simulated at each of 47 narrow-sense heritability values h^2^ ∈ {0.0002, 0.0004, 0.0006, 0.0008, 0.0010, 0.0012, 0.0014, 0.0016, 0.0018, 0.0020, 0.0022, 0.0024, 0.0026, 0.0028, 0.0030, 0.0032, 0.0034, 0.0036, 0.0038, 0.0040, 0.0042, 0.0044, 0.0046, 0.0048, 0.005, 0.006, 0.007, 0.008, 0.009, 0.01, 0.02, 0.03, 0.04, 0.05, 0.06, 0.07, 0.08, 0.09, 0.1, 0.2, 0.3, 0.4, 0.5, 0.6, 0.7, 0.8, 0.9}. At each heritability, standardized normal residuals (μ = 0, σ^2^ = 1) were drawn for each genotype class at the causative marker, and the mean of one class was shifted so that the genotype explained h^2^ of the total phenotypic variance. This yielded 47,000 single-QTL phenotypes in total (1,000 markers × 47 heritability values). The empirical 5% genome-wide LOD significance threshold was determined from 1,000 null phenotypes drawn from a standard normal distribution (h^2^ = 0) on the same 9,351 strains, taking the 95th percentile of the per-phenotype genome-wide maximum scaffolding marker LOD (LOD = 7.02).

### Calculation of LOD scores in QTL mapping

For each phenotype, at each tested marker, the LOD score was calculated as log_10_ L(B) − log_10_ L(A), where L(A) is the maximized likelihood of the data under a Gaussian null model with a single overall mean and L(B) is the maximized likelihood under a Gaussian alternative model with a separate mean estimated for each of the eight founder-haplotype classes defined by the segregant haplotype at that marker.

### Single-marker deviations from expected haplotype frequencies

**Fig. 2.**
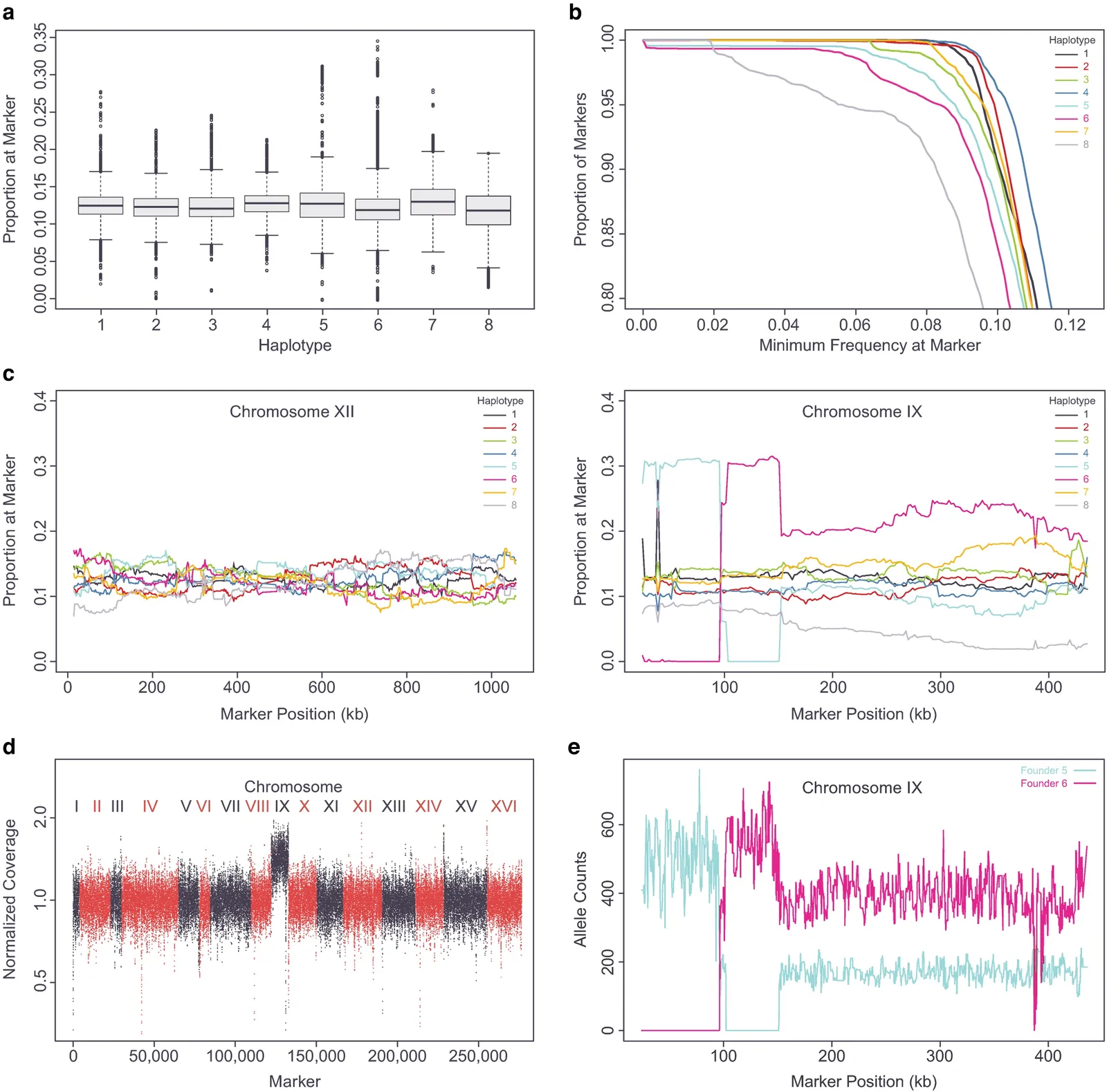
Genome-wide haplotype representation. a) Boxplot of haplotype proportion at each of the ∼5.5k scaffolding markers. b) Proportion of scaffolding markers with representation of each haplotype above cutoff. c) Haplotype proportion by scaffolding marker position across Chromosomes XII and IX. d) Genome-wide sequencing depth in haploid population G_1_5–G_1_6 measured in 31-bp sliding windows, normalized by genome median. Chromosomes indicated in alternating red and black. e) Allele counts on Chromosome IX, at markers polymorphic between Founders 5 and 6, from genome-wide sequencing of haploid population G_1_5–G_1_6.

Significant deviations from expected haplotype frequencies at the single-marker level were identified as follows among the haploid mapping strains. At each marker (except for those on Chromosome IX), the expected proportion of each haplotype in the mapping population is one-eight. Because of the extra copy of Chromosome IX in population G_1_5–G_1_6 (Figs. 1 and 2c, d, and e), expected haplotype frequencies are different for markers on that chromosome. Specifically, the expected frequencies of Haplotypes 1, 2, 3, 4, 7, and 8 are one-ninth for all Chromosome IX markers, while the expected frequencies of Haplotypes 5 and 6 vary between 0, one-ninth, two-ninth, and three-ninth, depending on the region (due to several LOH events on Chromosome IX that occurred along with the increase in chromosome copy number).

We compared the observed haplotype counts at each marker to these expectations using the Chi-square goodness-of-fit test. For its null distribution, this test assumes a Poisson distribution of counts around a mean equal to the expected count (in our case, usually one-eight of the total number of strains). In the Poisson distribution, the mean is equal to the variance. However, because our final mapping population is much larger than the intermediate cross populations (Supplementary Fig. 1), we anticipated that the count variance in the mapping population would be inflated relative to the mean, producing inflated Chi-square and significance values from the Chi-square test. To test this, we examined the central 95% of ordered counts, which should reflect the null distribution under the assumption that sites under selection are rare. A Q–Q plot of these counts against a simulated Poisson distribution with the same mean confirmed a linear relationship but with a much higher variance in the real counts (Supplementary Fig. 2a). Based on this relationship, we identified the divisor (31) that best transforms the central 95% of ordered counts back to a Poisson distribution (slope closest to 1 for regression against a simulated Poisson distribution) (Supplementary Fig. 2b).

The adjusted counts were used to calculate a *P*-value for goodness-of-fit at all scaffolding markers. The Holm method was then used to calculate a multiple-hypothesis corrected 0.05 *P*-value cutoff from these nominal values.

### Pairwise deviations from expected haplotype frequencies

The Chi-square test of independence was used to identify pairs of markers with significant deviation from haplotype independence. Because of the potential for the cross structure to inflate the Chi-square statistic, we examined the bottom 95% of observed interchromosomal Chi-square values, which should reflect the null distribution under the assumption that nonindependence between (unlinked) markers is rare. A Q–Q plot of these counts against a simulated Chi-square distribution with the same degrees of freedom confirmed that (null) Chi-square values are indeed elevated in our data (Supplementary Fig. 3). The strong log-linear relationship allowed us to transform the data back to the scale of the expected null distribution. A separate log-linear fit was used for pairwise interactions involving Chromosome IX (Supplementary Fig. 3).

A Chi-square value corresponding to a multiple-hypothesis corrected 0.05 *P*-value cutoff was then calculated from the transformed interchromosomal pair data using the Holm method.

## Results

### Choice of founder strains

To maximize the genetic diversity of our mapping population, we chose eight haploid “founder” strains isolated from diverse geographic and environmental sources (Fig. 1a and Table 1), with an emphasis on less extensively studied, non-European isolates. Four of these strains were isolated in China, where *Saccharomyces cerevisiae* originates and where genome diversity is greatest (Wang et al. 2012; Duan et al. 2018; Peter et al. 2018). Three of the Chinese strains were isolated from primordial forests (tropical, subtropical, and temperate) and one from a persimmon orchard. Among the non-Chinese strains, one was isolated from soil in the United States, one from soil in South Africa, one from Nigerian palm wine, and one from coconut wine in the Philippines (Table 1). WGS of these eight founder strains showed that they capture a large fraction of the currently known genetic diversity in the global population of *S. cerevisiae* (Materials and Methods) (Peter et al. 2018), including 32% of biallelic SNVs with a MAF greater than 0.005 and 56% of biallelic SNVs with a global MAF greater than 0.05.

**Table 1.** *S. cerevisiae* founder strains used in this study.

Strain name	Mating type	Derived from	Geographic origin	Reference	Genotype
YO2685 (Founder1)	MAT**a**	YO2681	Philippine tuba (coconut sap wine)	This study	*hoΔ1198::hisG*, *PHO87::hphMX6::ARS314*
YO2545 (Founder2)	MATα	YO2478	Cahokia, Illinois, USA, soil near oak tree	This study	*hoΔ1198::hisG*, *PHO87::natMX6::ARS314*
YO2563 (Founder3)	MAT**a**	YO2506	Aba, Abia State, Nigeria, Africa Raphia palm wine	This study	*hoΔ1198::hisG*, *PHO87::hphMX6::ARS314*
YO2552 (Founder4)	MATα	YO2485	Wuzhi Mountain, Hainan Island, China rotten wood in primeval, tropical forest	This study	*hoΔ1198::hisG*, *PHO87::natMX6::ARS314*
YO2633 (Founder5)	MAT**a**	YO2495	Wuyi Mountain Fujian, China Bark of *Hovenia acerba* (Chinese raisin tree), subtropical primeval forest	This study	*hoΔ1198::hisG*, *PHO87::hphMX6::ARS314*
YO2547 (Founder6)	MATα	YO2480	South Africa, Africa soil sample	This study	*hoΔ1198::hisG*, *PHO87::natMX6::ARS314*
YO2553 (Founder7)	MAT**a**	YO2487	Changping, Beijing, China persimmon orchard	This study	*hoΔ1198::hisG*, *PHO87::hphMX6::ARS314*
YO2588 (Founder8)	MATα	YO2492	Qinling Mountain, Shaanxi, China Bark of *Quercus fabrei* (oak) tree, temperate primeval forest	This study	*hoΔ1198::hisG*, *PHO87::natMX6::ARS314*

Using the founder WGS data, we identified 276,774 high-quality biallelic SNVs among the founder strains, a density of ∼1 SNVs per 44 bases, with only 0.031% of neighboring SNVs separated by more than 2 kb (File #1 in https://doi.org/10.5281/zenodo.17362653). Among these SNVs, 77.4% of minor alleles are unique to a single founder strain, 13.0% are shared by two founders and 6.73% by three founders, and 2.8% show a 4:4 distribution.

### Mapping population construction

To construct our mapping population, we used an eight-parent funnel cross with three rounds of mating (Fig. 1b). To initialize the cross, haploid *hoΔ0* founder strains were generated from the original diploid strains, with four MAT**a** and four MATα isolates chosen as founders (Materials and Methods). We avoided synthetic deletions of metabolic genes (auxotrophic markers) in the founder strains as these can have unintended effects on a range of phenotypes and tend to dominate QTL landscapes, confounding detection of naturally occurring variation (Brem et al. 2002; Warringer et al. 2011). Instead, drug resistance genes were inserted close to the mating type locus, encoding HYG resistance in the MAT**a** founders and NAT resistance in the MATα founders. These markers enabled haploid strains of each mating type to be identified at each step in the funnel cross and allowed diploid strains to be distinguished from haploids, facilitating mating (Materials and Methods).

The mapping population was derived from the eight founder haploids through three consecutive rounds of mating and sporulation as outlined in Fig. 1b with details in Supplementary Fig. 1. The second and third rounds of mating were carried out in pools, each consisting of 576 strains of a single mating type derived from spores isolated using a FACS-based approach (Sirr et al. 2022) that enables meiotic progeny to be isolated at scale (Materials and Methods). We used large pool sizes to prevent bottlenecking at intermediate steps of the funnel, maintaining genetic diversity and avoiding population structure in the final mapping population. At the end of the funnel process, 5,742 MAT**a** haploid cells and 5,742 MATα haploid cells were collected. Thus, the entire mapping population (prior to filtering for sequencing coverage) consists of 11,484 haploid strains, each a recombinant mosaic of the eight founder haplotypes. Unlike mice or other obligate diploid organisms, these strains did not require further inbreeding to achieve homozygosity.

### Individual strain ploidy and haplotype

Each of the 11,484 strains in the mapping population underwent low-coverage (median 3.8-fold) whole-genome sequencing (Materials and Methods). Strains with insufficient coverage (≤12k reads, approximately 0.1-fold coverage) were then removed, leaving 11,392 successfully sequenced strains in the mapping population. For these strains, we then used relative depth of sequencing coverage per chromosome to estimate copy number (Supplementary Table 2). This allowed us to identify aneuploid strains (Materials and Methods), which were set aside at this point (see below for discussion of this population), leaving 9,351 euploid (haploid) strains.

Genetically, each strain in the mapping population is a recombinant mosaic of the eight founder haplotypes. We inferred this recombinant haplotype for each euploid mapping strain using the sequencing data as follows. First, the number of reads supporting each of the two possible alleles at the ∼270k biallelic SNVs identified in the founder population was counted (Files #2 and #3 in https://doi.org/10.5281/zenodo.17362653). Then, a HMM was applied to these counts, with eight states representing the eight founder haplotypes. In the model, the alleles present in each of the founder strains were used to set allele emission probabilities for the reads at each SNV. Both estimated linkage between adjacent SNVs and the structure of the funnel cross were used to set transition probabilities between haplotype states (Materials and Methods). Applying this model allowed us to infer the most likely founder haplotype (encoded as values 1:8) of each mapping strain at each SNV position (File #4 in https://doi.org/10.5281/zenodo.17362653). Once a haplotype call has been made, the corresponding genotype follows directly from the founder allele at that position. We note that these haplotype calls are probabilistic rather than deterministic: each call is the maximum-likelihood founder assignment at that marker under the HMM, and haplotype–transition boundaries (crossover locations) carry additional uncertainty over a region of typically a few markers.

### Defining a ∼5,500 “scaffolding” marker subset

We next used the haplotyping data to assess three parameters of the euploid mapping population. First, we assessed genome-wide haplotype representation, quantifying how well the genetic diversity of the founder strains is maintained at each genomic position in the mapping population. Second, we assessed linkage between genomic regions, allowing us to identify any large-scale rearrangements in the founder strains that would disrupt marker order relative to the reference (S288c) genome. Finally, we assessed the extent of shared intermediate strain ancestry among the mapping strains, quantifying the degree of population structure. To carry out these analyses, we identified a “scaffolding” subset of 5,479 markers, spaced approximately 2 kb apart, drawn from the full set of ∼270k markers (File #5 in https://doi.org/10.5281/zenodo.17362653). This scaffold densely and evenly samples the genome but greatly reduces the number of markers that need to be assessed statistically, relative to the full set of ∼270k SNVs. Excluding the telomeric regions beyond the first and last marker on each chromosome, 97.7% of the genome is within 2 kb of a scaffolding marker.

### Haplotype representation

The proportion of mapping population strains with each founder haplotype was calculated for each of the scaffolding markers. As expected, the mean proportion for each haplotype genome-wide closely approximated 12.5% (one-eight) (Fig. 2a), with standard deviations ranging from 1.7% (Haplotype 2) to 3.0% (Haplotype 6). For every haplotype, representation at a level greater than 5% was seen across >95% of the genome (Fig. 2b).

Haplotype frequencies across a typical chromosome (Chromosome XII) are shown in Fig. 2c. In contrast, an unusual distribution of haplotype frequencies was seen across Chromosome IX (Fig. 2c), with an ∼50% elevated frequency of Haplotypes 5 or 6 relative to other haplotypes, one region with complete loss of Haplotype 5 and another region with complete loss of Haplotype 6. Pooled sequencing of haploid population G_1_5–G_1_6, formed by crossing Founders 5 and 6 (Fig. 1b), identified a 50% increase in the copy number of Chromosome IX relative to the rest of the genome (Fig. 2d; Materials and Methods). In addition, in this pooled sequencing, a 2:1 ratio of Haplotype 6 to Haplotype 5 was seen across most of the chromosome, along with two regions showing loss of either Haplotype 5 or Haplotype 6 representation (Fig. 2e), matching the pattern in the final mapping population (Fig. 2c). Haploid founder strains 5 and 6 are euploid; therefore, our data are consistent with a duplication of the Haplotype 6 copy of Chromosome IX in the diploid formed by mating them together (“DA3” in Supplementary Fig. 1), ie Trisomy IX, along with two LOH events on the left arm of Chromosome IX. This extra copy of Chromosome IX at the top of the funnel cross, made up of Haplotypes 5 and 6, explains the elevated frequency of these Chromosome IX haplotypes in the final euploid mapping strains (Fig. 2c), as well as the high frequency of Disomy IX among the aneuploid strains (see discussion of the aneuploid strains, below).

### Linkage behavior

**Fig. 3.**
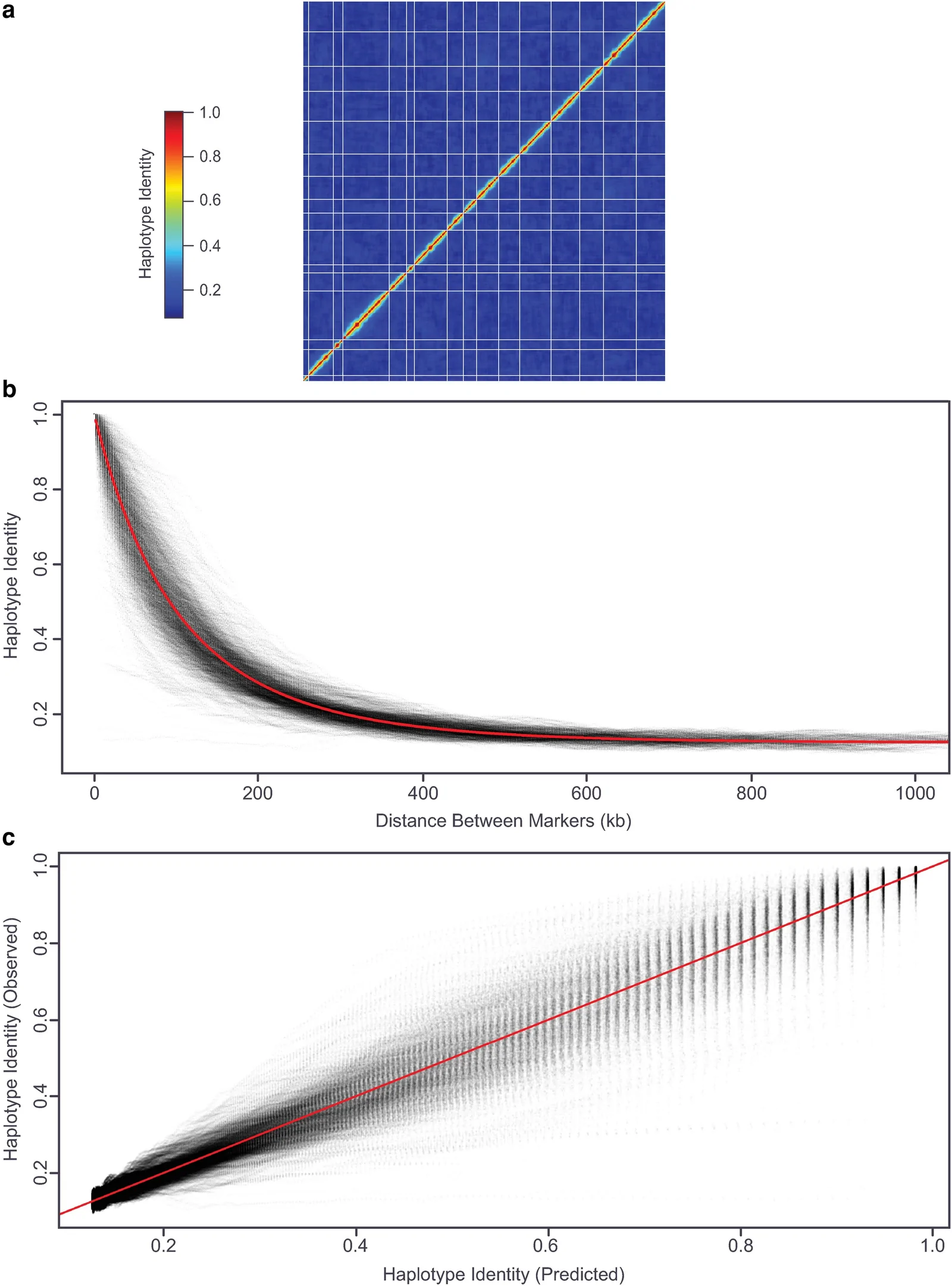
Genome-wide linkage behavior. a) Genetic linkage as proportion haplotype identity between each pair of scaffolding markers ordered from beginning of Chromosome I to end of Chromosome XVI. White lines separate chromosomes. b) Observed (gray) and fitted (8.7 crossovers per megabase, red) relationship between physical distance and linkage for each pair of scaffolding markers on the same chromosome. c) Observed linkage vs fitted linkage (based on 8.7 crossovers per megabase) for each pair of scaffolding markers on the same chromosome.

To estimate genetic linkage between genomic regions in the euploid population, for each of the scaffolding markers, the recombinant haplotype fraction (proportion of non-matching haplotypes) was calculated against all of the remaining markers. The resulting data (Fig. 3a) show little evidence of linkage patterns other than those expected by physical proximity between markers on the reference genome. That is, the marker linkage patterns are consistent with the S288c reference, indicating no large-scale rearrangements in the founder strains that would disrupt marker order. The only exceptions involve a small number of telomeric markers.

We also modeled the relationship between physical and genetic distances, assuming a Poisson distribution of crossovers (no interference) and a linear relationship between physical distance and the mean number of crossovers (genetic distance) (Materials and Methods). A genome-wide average value of 8.7 crossovers per megabase (after three rounds of meiosis) provided the best fit (Fig. 3b and c).

### Population structure

Population structure has the potential to generate spurious genotype–phenotype correlations. Our funnel cross was designed to minimize such structure in our mapping resource by using large pools of strains in the intermediate crossing steps, avoiding bottlenecking. However, processes such as variations in mating efficiency or growth could still lead to some degree of bottlenecking, with specific intermediate cross strains, and their associated haplotypes, contributing disproportionately to the final mapping population. Therefore, to quantify population structure and bottlenecking, for each pair of strains in the final (euploid) mapping population, we determined if they shared an ancestral strain in each of the intermediate haploid populations involved in the funnel cross.

A standard kinship matrix, as commonly used in multiparent QTL mapping, provides a single per-pair relatedness scalar but cannot directly attribute shared ancestry to specific intermediate populations within the funnel cross. To detect bottlenecking at each intermediate population separately, we used an intermediate-specific haplotype–identity analysis. For each pair of strains, we restricted the comparison to the regions of the genome both strains inherited from a particular intermediate population and then measured haplotype identity within that mask. Repeating this for each intermediate population identified pairs that shared ancestry through a member of that population.

Our approach is based on identifying regions of shared intermediate population ancestry in each pair of strains. For each strain in the final mapping population, the haplotype of each marker identifies the inheritance path of that region through the funnel cross structure. For example, any marker with Haplotype 1 was inherited from Founder 1, via intermediate haploid populations G_1_1–G_1_2 and G_2_1–G_2_4 (Fig. 1b). Similarly, each intermediate haploid population is responsible for inheritance of a specific set of founder haplotypes in the final progeny strains. For example, in every progeny strain, all markers with Haplotypes 1–4 were inherited via intermediate population G_2_1–G_2_4 (Fig. 1b), and all markers with Haplotypes 1–2 were inherited via intermediate population G_1_1–G_1_2 (Fig. 1b). In each progeny strain, this allows us to identify “G_1_1–G_1_2 inherited,” “G_2_1–G_2_4 inherited,” etc. regions.

Having identified regions in every strain descended from each intermediate population (eg population G_1_1–G_1_2), for each pair of strains we can then identify the common regions derived from that population in both strains. If the two strains share a single ancestor in the intermediate population of interest, their haplotypes will be identical across the shared region. Otherwise, for unrelated strains, the proportion of identical haplotypes in the shared region should be 0.5 (for intermediate “G_1_” populations consisting of two haplotypes; Fig. 1b) or 0.25 (for intermediate “G_2_” populations consisting of four haplotypes; Fig. 1b). For each pair of strains, and for each intermediate population, we quantified this as a discordance “distance” (proportion non-matching haplotypes in the shared region, Materials and Methods).

**Fig. 4.**
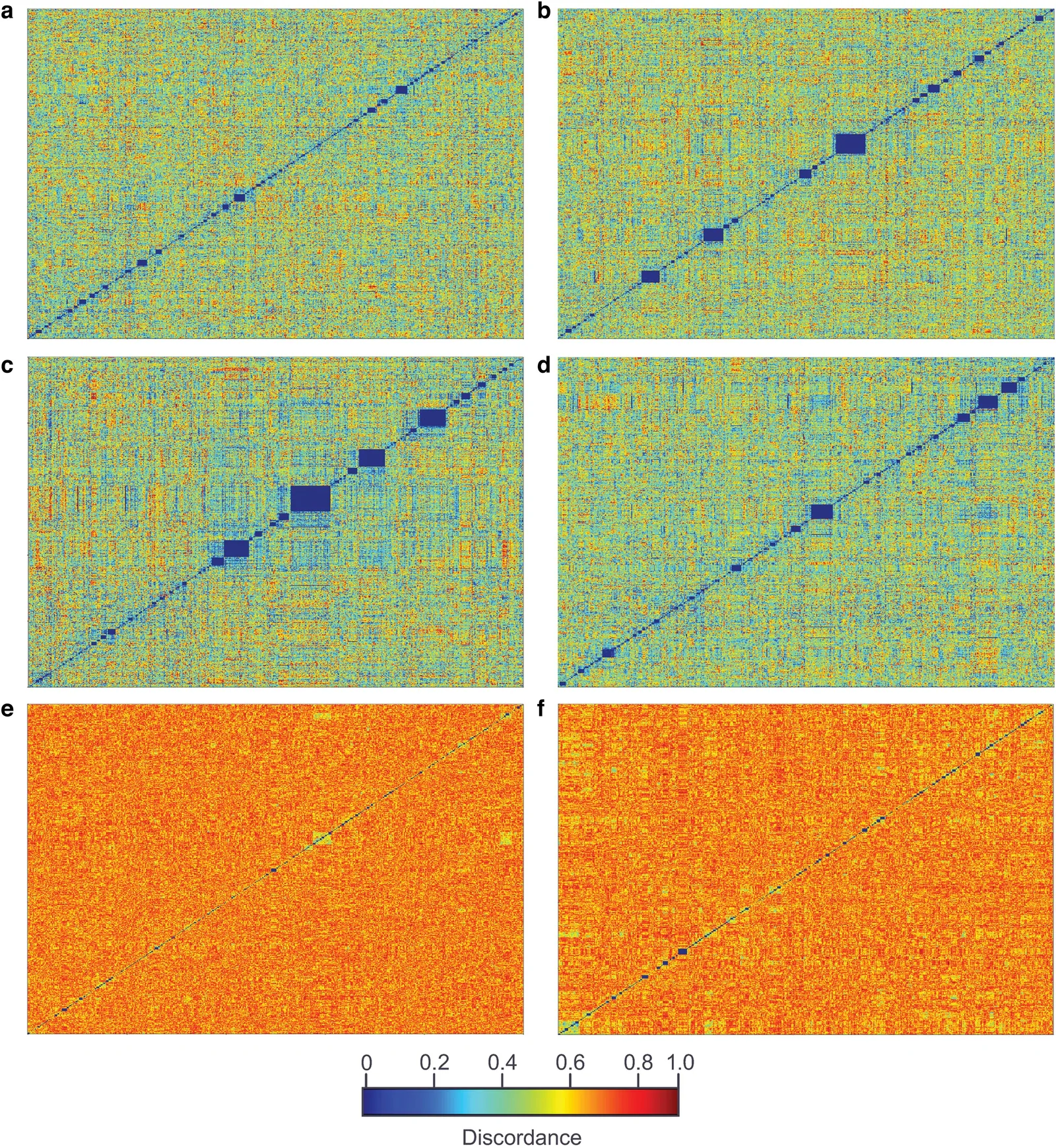
Quantifying shared ancestry in intermediate cross populations. All progeny strains clustered by pairwise haplotype discordance relative to each intermediate population. Shared ancestry is expected to produce a discordance value of 0. a) Population G_1_1–G_1_2. b) Population G_1_3–G_1_4. c) Population G_1_5–G_1_6. d) Population G_1_7–G_1_8. e) Population G_2_1–G_2_4. f) Population G_2_5–G_2_8. The discordance scale applies to all plots in the figure.

Clustering the results for each of the intermediate haploid populations (Materials and Methods) reveals only a few small clusters with shared ancestry and, therefore, little evidence of bottlenecking at the level of the “G_1_” or “G_2_” intermediate populations (Fig. 4).

These results both quantify the ancestral relationship between strains in the mapping population and demonstrate that the degree of population structure in our mapping population is very low.

### Scaffolding markers as an efficient and highly powered resource for linkage mapping

**Fig. 5.**
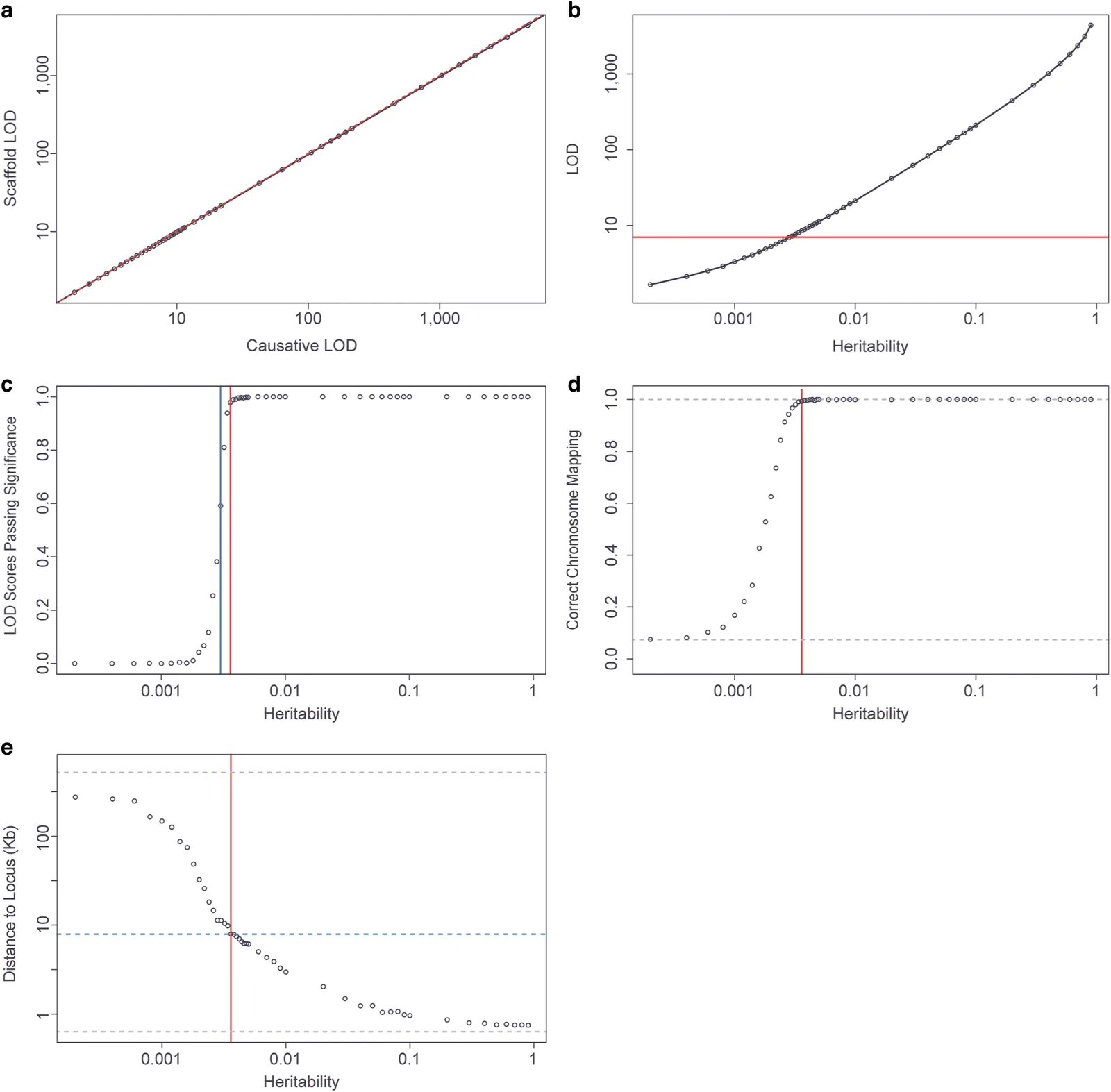
Simulated QTL mapping power and resolution. a) Mean LOD scores of causative markers vs most closely linked scaffold markers. Regression line in black, dashed 1:1 line in red. b) Causative marker heritability vs mean LOD at the most closely linked scaffolding marker. Red line indicates the 5% Type I family-wise LOD error threshold. c) Relationship between heritability at the causative marker and the proportion of LOD scores exceeding the 5% significance threshold at the most closely linked scaffolding marker. Blue and red lines, respectively, identify the heritabilities (0.3 and 0.36%) at which ≥50% and ≥95% of scaffolding markers exceed the significance threshold. d) Proportion of highest-scoring scaffolding markers mapped to same chromosome as causative maker, as a function of causative marker heritability. Lower gray dotted line indicates the proportion expected by chance (minimum possible) and the upper dotted line lies at 1 (maximum possible). Red line represents heritability of 0.36%. e) Mean distance between causative locus and highest-scoring scaffolding marker when on the same chromosome for different levels of heritability. Lower gray dotted line indicates the best possible resolution (distance from causative marker to most closely linked scaffold marker), and the upper dotted line indicates the worst possible resolution (mean distance between causative marker and a random marker on the same chromosome). Red line represents heritability of 0.36%, and blue dotted line represents mean distance between the maximal scaffolding marker and the causative marker for that heritability.

The ∼5.5k set of scaffolding markers was chosen to cover the yeast genome evenly and densely, such that 97.7% of the genome (excluding telomeric regions) is within 2 kb of a scaffolding marker. Therefore, at least one scaffolding marker should be closely linked genetically to any causative locus, and testing for QTL via an eight-level regression of phenotype on inferred founder haplotype (Materials and Methods) at each scaffolding marker should be an efficient, but still highly powered, alternative to applying the same test at every position in the full set of ∼270k high-quality SNVs. To compare the mapping power of the scaffolding set to that of the full set, we used a simulation framework based on independent single-QTL phenotypes. A panel of 1,000 causative markers was selected from the full ∼270k set (Materials and Methods). For each of these markers, we simulated one phenotype at each of 47 heritability values spanning h^2^ = 0.0002 to 0.9 (full grid in Materials and Methods), so that the single marker explains h^2^ of the total phenotypic variance, with the remainder being independent normal noise (mean 0, variance 1). This produced 1,000 × 47 = 47,000 single-QTL phenotypes in total, generated on the 9,351 euploid mapping strains. For each phenotype, we calculated LOD scores at the causative marker and at the scaffolding marker most closely genetically linked to it, regressing phenotype on haplotype (an eight-level factor) at each marker (Materials and Methods). Across all heritability levels, the linked scaffolding markers showed very little reduction in power relative to the causative loci, with a regression estimate of LOD_scaff_ = LOD_caus_^0.9949^ (Fig. 5a).

### Predicted power and mapping resolution

Based on the similarity in power between the full set of markers and the scaffolding subset, we used the scaffolding markers to assess the power of our mapping population to detect a single causative QTL. We first established an empirical 5% genome-wide Type I error threshold. Specifically, we generated 1,000 null phenotypes (each sampled from a standard normal distribution with mean 0 and variance 1, ie h^2^ = 0) on the 9,351 euploid mapping strains, calculated LOD scores at every scaffolding marker, and recorded the maximum LOD score per phenotype. The 95th percentile of these per-phenotype maxima defined the empirical 5% genome-wide significance threshold (LOD = 7.02).

Returning to the 47,000 single-QTL simulated phenotypes described above, we then carried out a genome-wide regression scan across all scaffolding markers for each phenotype and identified the marker with the highest genome-wide LOD score. We refer to this maximum LOD scaffolding marker as the “candidate marker” for that phenotype. Because each phenotype is driven by a single causal locus, this candidate–marker statistic measures the power to detect that one underlying QTL. The mean candidate–marker achieved genome-wide significance (LOD > 7.02) for phenotypes with h^2^ ≥ 0.003 (Fig. 5b). The median candidate–marker LOD also achieved significance at this level of heritability, while at h^2^ ≥ 0.0036 more than 95% of individual candidate–marker LOD scores exceeded the genome-wide threshold (Fig. 5c). Together, these results indicate that the scaffolding markers are highly powered to detect single QTL with h^2^ ≥ 0.0036.

To assess the mapping resolution provided by the scaffolding marker set, we compared the position of each candidate marker to that of the underlying causative locus. Consistent with the empirical significance cutoff, the frequency with which the candidate marker lies on the same chromosome as the causative locus declines rapidly at heritabilities below 0.003 (Fig. 5d). At our more highly powered heritability cutoff of 0.0036, the candidate marker is on the correct chromosome 99.3% of the time (Fig. 5d), with a mean distance to the causative locus of 7.9 kb (Fig. 5e). This distance falls below 5, 2, and 1 kb at heritabilities ≥ 0.007, 0.03, and 0.09, respectively (Fig. 5e).

Together, these results demonstrate that mapping with the scaffolding markers is highly powered to detect single QTL with h^2^ ≥ 0.0036 and to resolve the position of causative loci to a few kilobases or less.

### Loci with single and pairwise deviations from expected haplotype frequencies

**Fig. 6.**
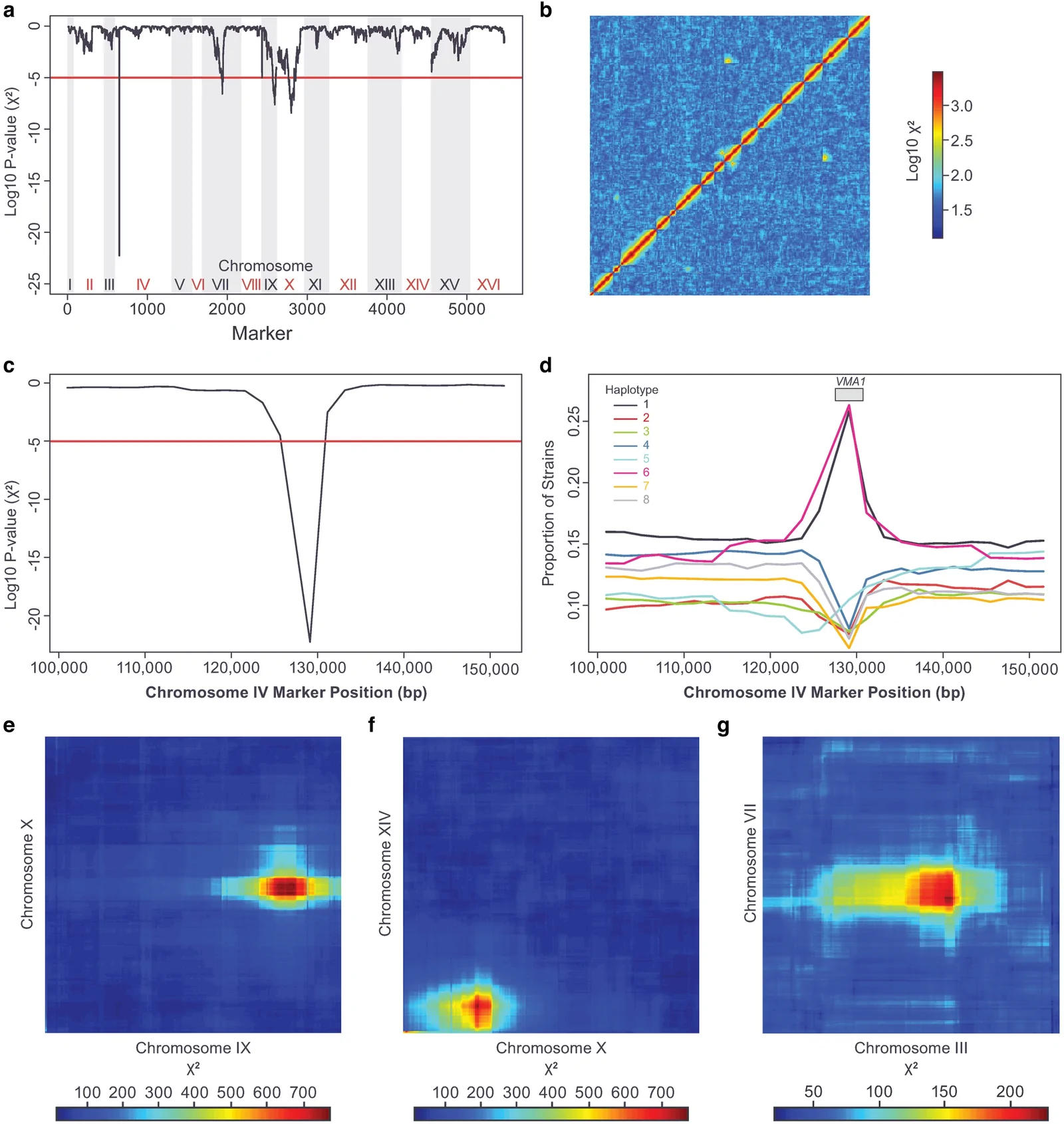
Single-marker and pairwise deviations from expected haplotype frequencies. a) Nominal Chi-square *P*-value for single-marker deviation from expected haplotype proportions. Multiple-hypothesis corrected 5% significance cutoff (Holm method) shown in red. Chromosomes indicated with alternating gray and white background. b) Log_10_ Chi-square statistic values for pairwise independence between markers genome-wide. Multiple-hypothesis corrected 5% significance cutoff (Holm method) corresponds to a value of 131 (log_10_ value = 2.12). c) Close-up of panel a) for region on Chromosome IV around VMA1 gene. d) Haplotype proportions for region on Chromosome IV around VMA1 gene. (e–g) Chi-square statistic values for pairwise independence between markers on the two chromosomes. Multiple-hypothesis corrected 5% significance cutoff (Holm method) is equal to 131.

The repeated steps of mating, sporulation, and growth that make up the funnel cross process have the potential to act as a selective regime, enriching for high-fitness alleles and their associated haplotypes and depleting low-fitness alleles and haplotypes. These processes would produce deviations from the background distribution of haplotype frequencies at the single-marker level. Similarly, pairwise interactions between loci could also affect fitness, including through synthetic inviability. Such pairwise interactions would generate statistical nonindependence between haplotypes at the two interacting loci. We used the scaffolding markers to identify loci where haplotype frequencies deviate significantly from the null expectation for single markers (generally one-eight proportion of each haplotype, Materials and Methods). In addition, we also looked for significant levels of haplotype nonindependence between all pairs of scaffolding markers (Materials and Methods). These analyses identified a strong signal at the single-marker level on Chromosome IV (Fig. 6a) as well as three sets of strong pairwise interactions (Fig. 6b), which also had marginal effects detectable at the single-marker level (Fig. 6a).

The single-marker signal on Chromosome IV (Fig. 6a) localizes to the *VMA1* gene (Fig. 6c) and is caused by a spike in the frequency of Haplotypes 1 and 6 over a short region centered on the gene (Fig. 6d). This pattern is the expected result of a known gene conversion process driven by a homing endonuclease. Specifically, the *VMA1* sequences of founder strains 1 and 6 encode the site-specific homing endonuclease PI-SceI, while the other six founders have *VMA1* sequences that lack the endonuclease sequence (Supplementary Fig. 4). PI-SceI is released from Vma1 by protein splicing, and, during meiosis, PI-SceI cleaves *VMA1* sequences that lack the endonuclease sequence, initiating conversion to the endonuclease-containing form via recombination (Gimble and Thorner 1992). Our data are consistent with this process occurring in the intermediate funnel cross diploids (DA1, DA3, DB1-4, DC1-2; Supplementary Fig. 1) where an allele of *VMA1* containing the endonuclease (from Founders 1 or 6) is present with an endonuclease-free *VMA1* gene derived from any of the other founders.

Three pairs of loci displayed strong and significant deviations from haplotype independence (Materials and Methods). For the first of these pairs, the maximal deviation was for scaffolding markers at positions 361,254 on Chromosome IX (in the region of the *AIM1* gene) and 357,553 on Chromosome X (in the region of the *SDH1* gene) (Fig. 6e). Examination of the pairwise haplotype count table revealed a “synthetic viability” pattern for Haplotype 8, where there are almost no strains with Haplotype 8 at either locus except when both loci have Haplotype 8 (Supplementary Table 5). This pattern suggests inviability when Haplotype 8 at either locus is combined with any other haplotype at the other locus. The second pairwise interaction was maximal between marker pairs at positions 204,464 on Chromosome X (in the region of the *MDV1* gene) and 116,372 on Chromosome XV (in the region of the *NDJ1* gene) (Fig. 6f) and showed the same kind of “synthetic viability” pattern seen with the first pair of loci, but this time involving Haplotype 6 (Supplementary Table 6). The final pairwise interaction was maximal for markers at positions 201,158 on Chromosome III (adjacent to the mating type locus) and 481,269 on Chromosome VII (in the region of the *PMA1* gene) (Fig. 6g). This pairwise interaction displayed a more complex pattern than that seen with the two “synthetic viability” interactions (Supplementary Table 7).

Depending on the pattern of interaction, pairwise deviations from haplotype independence can, but do not always, produce marginal haplotype effects detectable at the single-marker level. The three pairwise interactions described here colocalize with the significant deviations from expected haplotype frequencies at the single-marker level on Chromosome VII, IX, and X (Fig. 6a and b).

### Aneuploid strains

Finally, we analyzed the aneuploid subpopulation of mapping strains that were separated from the euploid strains earlier in our analysis. Aneuploidy occurs frequently in wild strains and is often well tolerated (Hose et al. 2015; Peter et al. 2018; Scopel et al. 2021), and aneuploid strains were quite common among the progeny of the funnel cross, consisting of 2,041 strains (17.9% of the total mapping population, after removing strains with low sequencing coverage). Relative chromosome coverage indicated which chromosomes were disomic in each strain, ie no chromosome copy numbers higher than 2 were observed (Supplementary Table 2). Haplotypes were inferred for monosomic chromosomes as described for the euploid subpopulation, while a separate model was used to infer the haplotypes of disomic chromosomes (Materials and Methods). The final haplotypes for each aneuploid strain are given in File #7 in https://doi.org/10.5281/zenodo.17362653.

Among the 2,041 aneuploid strains, 1,820 (89.2%) displayed patterns of sequence coverage consistent with disomy of a single chromosome, and 206 (10.1%) had disomy of two chromosomes (Supplementary Table 2). Only 15 (0.7%) of strains had disomy of three or more chromosomes. Although disomies of all chromosomes were observed in the aneuploid population, the most common were of Chromosomes IX (49.7%), X (16.5%), and I (14.5%). The full per-chromosome disome counts were I, 332; II, 49; III, 22; IV, 8; V, 48; VI, 1; VII, 14; VIII, 68; IX, 1,135; X, 378; XI, 72; XII, 66; XIII, 21; XIV, 43; XV, 15; and XVI, 12. Chromosome I is the smallest yeast chromosome and frequently displays copy number changes in natural yeast isolates (Peter et al. 2018; Scopel et al. 2021) implying that it is prone to missegregation during cell division. Disomy I is also well tolerated (Torres et al. 2007; Scopel et al. 2021) likely because it perturbs the copy number of only a small proportion of the genome. The high observed frequency of Disomy IX is expected given the extra copy of this chromosome inferred to have been present in strain DA3 (Fig. 2c, d, e and Supplementary Fig. 1). Like Chromosome I, disomy of Chromosome IX is also frequently observed in strains not adapted to laboratory conditions (Zhu et al. 2016; Peter et al. 2018; Scopel et al. 2021). Disomy X co-occurs much more frequently with Disomy IX than expected by chance based on their individual frequencies in the full (∼11k) mapping population (Chi-squared test of independence: *P* < 1 × 10^−16^), with 30.7% of strains disomic for Chromosome X also disomic for Chromosome IX (Supplementary Table 2). The known mechanism giving rise to Disomy IX at the beginning of the funnel cross suggests that Disomy X arose preferentially from strains that were already disomic for Chromosome IX. This could occur in one of two ways. First, Disomy X could have arisen in a small number of ancestral strains disomic for Chromosome IX, followed by expansion (selective or stochastic) of these lineages. Second, Disomy X could have arisen independently multiple times in strains disomic for Chromosome IX, ie at an elevated rate in such strains. The first possibility would be detectable as strong bottlenecking in population G_1_5–G_1_6 (Fig. 1b) among the Chromosome X disomes, which is not observed (Supplementary Fig. 5). Therefore, it appears that Disomy X arose multiple times independently but was strongly associated with existing Chromosome IX disomy, suggesting that increasing the copy number of Chromosome X may be adaptive in strains with an extra copy of Chromosome IX.

The patterns of aneuploidy observed in CYClones share key features with those reported in natural *S. cerevisia*e populations but also reveal informative differences arising from the design of the cross. A unified analysis of 3,034 natural isolates (Loegler et al. 2024) reported aneuploidy in 31% of strains, with Chromosomes I, III, VI, and IX the most commonly disomic and an inverse correlation between aneuploidy frequency and chromosome length. Chromosome I, the shortest yeast chromosome, is correspondingly the most frequently disomic in this natural-population spectrum, consistent with its small size and high tolerability. In CYClones, Chromosome I disomy remains common (14.5% of aneuploids) but is only the third most frequent, behind Chromosome IX (49.7%) and Chromosome X (16.5%). This reordering is fully attributable to a single event in the funnel cross: the Trisomy IX inferred to have arisen in intermediate diploid DA3 (Fig. 2c, d, e; Supplementary Fig. 1). This event elevated the frequency of Chromosome IX disomy across the entire mapping population, and the nonrandom co-occurrence of the Chromosome X disomy described above produced a parallel elevation of Chromosome X disomy as a downstream consequence. However, even with this founder-driven enrichment of Chromosomes IX and X, the overall aneuploidy rate in CYClones (17.9%) is lower than that in the natural population (31%), consistent with the haploid ploidy of the CYClones recombinant strains, where aneuploidy is less tolerated than in the predominantly diploid strains surveyed in natural-population studies (Strope et al. 2015; Peter et al. 2018; Loegler et al. 2024).

## Discussion

CYClones, a library of 11,392 individually genotyped haploid segregants from an eight-parent funnel cross, is presented here as a resource for the yeast community. The founders were chosen to capture a large fraction of natural *S. cerevisiae* diversity (56% of biallelic SNVs at global MAF >5%) (Peter et al. 2018), and each segregant's haplotype, a mosaic of the eight founders, was inferred from low-coverage whole-genome sequencing at ∼270,000 SNV positions. The core of the mapping population consists of 9,351 euploid strains that pass sequence coverage filters, and these strains display the structure expected from the funnel design: linkage is consistent with the S288c reference coordinate system, with no large-scale rearrangements in the founder strains that would disrupt marker order; population structure is low, with no evidence of bottlenecking at any intermediate cross stage; and all eight founder haplotypes are represented at >5% across >95% of the genome. Only a small number of loci show significant deviations from expected allele frequencies, but these include a gene conversion process at the *VMA1* locus and several pairwise “synthetic viable” interactions.

CYClones was designed primarily as a powerful tool for QTL mapping, with the large size of the genotyped population and high density of markers providing statistical power to detect loci of small effect and gene–gene interactions. In addition, the density of recombination events associated with three rounds of crossing during strain construction should allow the positions of QTL to be mapped with high resolution. Furthermore, in the euploid population, we demonstrate that a 2 kb spaced set of ∼5,500 “scaffolding” markers represents an efficient way of harnessing this power relative to the full set of ∼270k markers, with 97.7% of the genome (excluding telomeric regions) lying within 2 kb of a scaffolding marker. Using simulated phenotypes affected by a single genetic locus, we demonstrate that the most closely linked scaffolding marker is almost equally powered to detect a QTL compared to the causative marker itself. Further simulations indicate that the scaffolding markers are highly powered to detect single QTL with $h^{2}\geq 0.36\text{\%}$ and to identify the location of such causative loci at a resolution of a few kb or less. Once a QTL has been detected using the scaffolding markers, its position can be further resolved using local markers drawn from the full set of ∼270k.

An end-to-end demonstration of CYClones in use is provided in a companion manuscript (Cromie *et al.*, bioRxiv https://doi.org/10.1101/2025.10.27.683165), in which the population is used to map QTL across 10 solid–medium growth conditions. The inferred founder haplotypes are also provided as a precomputed R/qtl2 founder–probability object (Broman et al. 2019), ready for direct use in QTL mapping (see File #8 in Data availability). To facilitate downstream identification of candidate causative variants at any region of interest identified through the CYClones linkage framework, including structural variants (which can have substantial phenotypic effects in yeast [Loegler et al. 2025]), we have additionally deposited near telomere-to-telomere assemblies of the eight founder strains together with SNV, small indel, and structural-variant call sets against S288c (R64-1-1), under the existing CYClones Zenodo record (File #9 at https://doi.org/10.5281/zenodo.17362653). These calls were not used to generate the marker set used in this study, which was generated from short-read sequencing matched to the short-read sequencing of the progeny strains.

In addition to the euploid core, we also identified a substantial subset of 2,041 CYClones strains containing a variety of disomic chromosomes. These aneuploid strains are fully haplotyped, including the disomic chromosomes. Unlike mammals, where an increase in copy number of most autosomes during meiosis is a fatal event, in yeast aneuploidy is commonly observed in strains isolated from the environment, agricultural products, and immunocompromised individuals (Strope et al. 2015; Gallone et al. 2016; Ludlow et al. 2016; Zhu et al. 2016; Duan et al. 2018; Peter et al. 2018; Fay et al. 2019; Scopel et al. 2021). Copy number changes in yeast can be an advantageous means of rapidly adapting to environmental stresses, undergoing phenotype switching, or balancing the effect of genetic incompatibilities (Rancati et al. 2008; Tan et al. 2013; Sirr et al. 2015). Like their euploid counterparts, the aneuploid subset of CYClones encompasses a large portion of the genetic variation known to exist in *S. cerevisiae*. Therefore, these segregants represent a singular resource for exploring interactions between copy number and allelic effects on phenotype. While the aneuploid population includes at least one strain (and generally more than a dozen strains) for each of the 16 possible disomies, a duplication event at the top level of the cross has resulted in >1,000 strains containing a Chromosome IX disomy, making this subset of aneuploids highly powered for characterizing QTL in the context of a specific disomic chromosome.

CYClones complements rather than replaces existing yeast mapping resources, with each resource (including CYClones) having particular strengths and weaknesses. Compared with natural-population GWAS panels (Peter et al. 2018; Loegler et al. 2024, 2025), CYClones offers a controlled pedigree largely free of the population–structure confounding and clonal-history linkage disequilibrium that complicates natural-isolate association mapping, at the cost of capturing a smaller fraction of the natural allelic diversity (eight founder genomes vs more than a thousand). Compared with large biparental crosses such as the BYxRM panel (Bloom et al. 2013) and the X-QTL pooled-segregant approach (Ehrenreich et al. 2010), CYClones samples a much wider slice of natural *S. cerevisiae* variation: ∼270,000 SNVs segregate in the eight-founder panel, well above the number segregating between any single pair of parents. As in a biparental cross, most of the CYClones variants are biallelic; unlike a biparental cross, the minor allele is typically present in only one or two of the eight founders, at ∼12.5 to 25% frequency in the segregating population rather than at ∼50%. This denser sampling of variation at lower per-variant frequency reduces per-locus power for any specific allele but allows QTL discovery and analysis across the broader genetic variation captured by the eight founders and reduces the dependence of any QTL discovery on the genetic background of a single pair of parents.

Among multiparent yeast resources, the closest design–space neighbor of CYClones is the 16-founder round-robin panel of (Bloom et al. 2019), which similarly uses individual whole-genome sequencing of ∼14,000 haploid segregants from a multiparent cross. However, in a typical CYClones segregant, the genome is a mosaic of haplotype segments drawn from across the eight founders, while in the round-robin design, by contrast, each segregant inherits alleles from only the two founders of its specific cross, and only 16 of the 120 possible founder pairs are directly crossed. The funnel cross design also gives more meiotic events per strain than a round-robin (∼9 crossovers per Mb over three rounds of meiosis, vs the single F1 round of a round-robin), supporting finer mapping resolution per causative locus. Other multiparent yeast populations are more distantly related: the four-parent SGRP-4X (Cubillos et al. 2013) uses fewer alleles, and the 18-way Linder *et al.* outcrossed populations (Linder et al. 2020) use 12 rounds of outcrossing and pooled rather than individual genotyping.

The scale that makes CYClones powerful for QTL mapping also imposes practical constraints on its use. Phenotypes must be assayable accurately and reproducibly in high throughput, making CYClones best suited for growth-based assays, fluorescence- and imaging-based screens, and any phenotype amenable to multi-well-plate or pin–tool measurement. Effective use of the resource at this scale requires the laboratory infrastructure to handle and phenotype thousands of strains in parallel.

Beyond QTL mapping, CYClones is well-suited to a range of other uses. Given the density of variant sites in the population and the multiple rounds of meiosis, our library provides a rich dataset for understanding the molecular mechanisms of recombination and chromosomal segregation. CYClones could also be used to explore the genome dynamics of nonreciprocal events associated with retrotransposition (Ty elements) or homing endonucleases, such as the effect of PI-SceI at the *VMA1* locus described in this study. The collection is also a powerful resource for identifying yeast strains with extreme phenotypes, with utility for industrial, pharmaceutical, and food science applications. In short, the CYClones collection is a powerful and flexible tool for the research community.

## Supplementary Material

jkag179_Supplementary_Data

## Data Availability

Sequence reads for the eight parental founder strains are deposited in the European Nucleotide Archive (ENA) (accession PRJEB80495), and sequence reads for the progeny strains are available under accession PRJEB72283. All supporting datasets are publicly available as supplementary files on Zenodo at https://doi.org/10.5281/zenodo.17362653 (CC BY 4.0 license): parent marker genotypes (File #1), raw marker read counts (Files #2 and #3), inferred euploid and aneuploid haplotypes (Files #4 and #7), the 2 kb marker scaffold (File #5), and an R analysis pipeline together with its raw input files (File #6 archive) that reproduces all analyses described in the Results. Full details of haplotype inference, including emission and transition probabilities and use of the Viterbi algorithm, are described in the Materials and Methods to enable independent reimplementation if desired. A precomputed R/qtl2 founder-call object (File #8: cyclones_genoprob.rds) derived from the CYClones haplotype calls (encoded as binary probability vectors) is also deposited, allowing it to be loaded with readRDS() and passed directly to R/qtl2's downstream QTL mapping functions without further reformatting. The same Zenodo record also includes a downstream T2T resource for causative-variant follow-up at any QTL region of interest (File #9). For each of the eight founders, the deposit provides a near telomere-to-telomere assembly, MUMmer4 and minimap2 alignments against S288c (R64-1-1), a SNV/small-indel VCF (<50 bp), a structural-variant VCF (≥50 bp) from MUM&Co, and a SyRI overlay of the same alignments. The SNV/indel and SV calls are also provided as multi-sample merged VCFs. Bash workflow scripts, a pinned conda environment specification (cyclones_sv.yaml) for cross-platform reproducibility, and a stand-alone concordance script comparing the per-founder paftools.js SNV calls to the short-read founder marker set are included, with a README documenting exact tool versions and command lines. The raw Nanopore reads underlying the T2T assemblies are deposited at the ENA (accession PRJEB114491). Strains are available directly from the corresponding author on request, on a cost–recovery basis covering materials, labor, and shipping. Supplemental material available at G3 online.
